# BACHD rats expressing full-length mutant huntingtin exhibit differences in social behavior compared to wild-type littermates

**DOI:** 10.1371/journal.pone.0192289

**Published:** 2018-02-07

**Authors:** Giuseppe Manfré, Arianna Novati, Ilaria Faccini, Andrea C. Rossetti, Kari Bosch, Raffaella Molteni, Marco A. Riva, Johanneke E. Van der Harst, Huu Phuc Nguyen, Judith R. Homberg

**Affiliations:** 1 Donders Institute for Brain, Cognition and Behaviour, Department of Cognitive Neuroscience, Radboud University Medical Center, Nijmegen, The Netherlands; 2 Noldus Information Technology BV, Wageningen, The Netherlands; 3 Institute of Medical Genetics and Applied Genomics, University of Tübingen, Tübingen, Germany; 4 Centre of Rare Diseases, University of Tübingen, Tübingen, Germany; 5 Department of Pharmacological and Biomolecular Sciences, University of Milan, Milan, Italy; 6 Department of Medical Biotechnology and Translational Medicine, University of Milan, Milan, Italy; Northeastern Ohio Medical University, UNITED STATES

## Abstract

**Background:**

Huntington disease (HD) is a devastating inherited neurodegenerative disorder characterized by progressive motor, cognitive, and psychiatric symptoms without any cure to slow down or stop the progress of the disease. The BACHD rat model for HD carrying the human full-length mutant huntingtin protein (mHTT) with 97 polyQ repeats has been recently established as a promising model which reproduces several HD-like features. While motor and cognitive functions have been characterized in BACHD rats, little is known about their social phenotype.

**Objective:**

This study focuses especially on social behavior since evidence for social disturbances exists in human patients. Our objective was to compare social behavior in BACHD and wild-type (WT) rats at different ages, using two different measures of sociability.

**Methods:**

Animals were tested longitudinally at the age of 2, 4 and 8 months in the social interaction test to examine different parameters of sociability. A separate cohort of 7 month old rats was tested in the three chamber social test to measure both sociability and social novelty. Gene expression analyses in 8 months old animals were performed by real time qRT-PCR to evaluate a potential involvement of D1 and D2 dopaminergic receptors and the contribution of Brain-derived neurotrophic factor (BDNF) to the observed behavioral alterations.

**Results:**

In the social interaction test, BACHD rats showed age-dependent changes in behaviour when they were-re introduced to their cagemate after a 24 hours-period of individual housing. The time spent on nape attacks increased with aging. Furthermore, a significant higher level of pinning at 2 months of age was shown in the BACHD rats compared to wild-types, followed by a reduction at 4 and 8 months. On the other hand, BACHD rats exhibited a decreased active social behaviour compared to wild-types, reflected by genotype-effects on approaching, following and social nose contact. In the three chamber social test, BACHD rats seemed to show a mild deficit in preference for social novelty, but no changes in social interest. Molecular analyses revealed that BACHD animals exposed to the social interaction test displayed decreased mRNA levels of the total form of BDNF in ventral striatum and unaltered striatal expression of D1 and D2 dopamine receptors.

**Conclusions:**

Taken together, these results indicate deficits in several parameters representative of sociability. Altered BDNF expression in the ventral striatum may contribute to the deficits in sociability in 8 months old BACHD rats. These data support the validity of the BACHD rat model in mimicking features of certain social deficits that could be relevant to symptoms in patients.

## 2 Introduction

Huntington disease (HD) is a dominantly inherited neurodegenerative disorder that is caused by an unstable expansion of a CAG repeat within the coding region of the huntingtin (*HTT*) gene [1]. It is characterized by motor impairment, abnormal choreic involuntary movements and by psychiatric, psychological and intellectual disorders [2]. Although more emphasis has been placed on detecting the early cognitive and motor impairments [3–6], emotional dysfunction might also precede the clinical HD diagnosis [7,8]. Thus, the identification of early psychiatric symptoms may be particularly important in HD because of their deleterious effects on everyday functioning and quality of life [8,9]. Characterizing early neuropsychiatric phenotypes in animal models of HD is therefore especially important.

The BACHD rat model of Huntington disease was generated using a human bacterial artificial chromosome (BAC) which contains the full-length *HTT* genomic sequence with 97 CAG/CAA repeats and all regulatory elements [10]. BACHD rats present motor, cognitive and emotional alterations. Previous characterization studies in these rats showed clasping behavior starting at the age of 3 weeks, robust deficits in the Rotarod task already at 1 month [10], decreased activity in the Phenomaster from 3 till 6 months [10] and mild gait alterations in the Catwalk test by 12 months of age [11]. In a fear conditioning set-up, BACHD rats exhibited associative memory deficits by 4 months of age while an impairment of their reversal learning performance emerged at 6 months when rats were tested in a cross maze task [12]. At this age, also signs of fronto-striatal impairment were observed in different Skinner box tasks for short term memory [13]. Starting at 4 months of age, BACHD rats display also changes in emotionality as suggested by the decreased anxious-like behavior in the elevated plus maze [10]. At a comparable age (between 3 and 5 months), an impulsive-like phenotype was demonstrated in a delayed discounting paradigm and in the Differential Reinforcement of Low Rate of Responding task [14] while later, at 9 months of age, deficits in prepulse inhibition became evident as well [11]. In spite of the extensive use of the BACHD rat line in the last years, its psychiatric phenotype has been only partly investigated indicating that the BACHD rat line requires further phenotyping.

In this study, we focused on social behavior of BACHD rats as its changes are an important component of neuropsychiatric symptoms. Some aspects of social behavior have been partly examined in different rodent models of HD, showing altered social interaction [15–17] and social preference [15], reduced social memory and recognition [18,19] and even increased levels of aggression [20], supporting the view of impaired social behavior in HD.

We therefore investigated different parameters of the social behavior repertoire and associated molecular alterations in the BACHD rat model of HD. We performed detailed analyses of social behavior parameters to monitor development of potential deficits over time. To achieve this aim we performed: 1) a social interaction test to investigate different parameters of sociability at different ages; 2) a so-called three chamber social test to assess both sociability and social novelty. To link behavior to molecular correlates of social behavior, we quantified mRNA levels of D1 and D2 dopamine receptors and the total form of BDNF. Subtle impairments in specific aspects of social behavior were found which could be relevant readouts in pre-clinical HD-treatment studies. Additionally, changes in molecular markers were detected which could underlie the social behavior deficits in HD.

## 3 Material and methods

### Ethical statement

The experiments were carried out at 2 different locations. The experiments reported here were either approved by the Animal Ethics Committee (Dier Experimenten Commissie, RUDEC, Nijmegen, The Netherlands) or by the local ethics committee (Regierungspraesidium Tuebingen, Germany), in full compliance with the European Union legislation on the use of animals for scientific purposes (Directive 2010/63/EU). All experimental procedures at Radboudumc (Nijmegen, The Netherlands) were performed under a project license from the Central Committee on Animal Experiments (Centrale Commissie Dierproeven, CCD, The Hague, The Netherlands), in full compliance with the legal requirements of Dutch legislation on the use and protection of laboratory animals (Animal Testing Act, WOD). All experimental procedures at the University of Tuebingen (Tuebingen, Germany) were performed in accordance with the German Animal Welfare Act and the guidelines of the Federation of European Laboratory Animal Science Associations.

### Animals

For the social interaction test, fifteen transgenic males were supplied from a BACHD colony (TG5 line, Yu-Taeger et al., 2012) at Charles River (Wilmington, MA, USA) and an in-house breeding colony was preserved and maintained at Radboudumc (Nijmegen, The Netherlands) by cross-breeding these males with wild-type female rats (Charles River, Germany). WT and BACHD animals (N = 24/group) were maintained on a Sprague-Dawley (SD) background. Genotyping and determination of BAC transgene integrity were performed via PCR analysis using genomic DNA extracted from ear biopsy tissue at postnatal day (PND) 21. Rats were weaned at PND 21 and test pairs were then group-housed two per cage with littermates of the same genotype and sex in a constant temperature (19.5 ± 1°C) and humidity room (55 ± 10%) with a reversed 12 h light/dark cycle with lights on/off at 8:00 P.M. /8:00 A.M.). Housing by test pairs (N = 2 siblings per cage) was chosen because two familiar animals were tested for social interaction, on the base of previously published protocols [21].

All experimental animals used in the three chamber social test (University of Tuebingen, Germany) were bred on an SD background by pairing heterozygous BACHD males with wild-type females (Charles River, Germany). Rats were genotyped following previously used protocols [10] at PND 20. At weaning (PND 21), rats were housed in groups of 4 with same sex-littermates of mixed genotype like in previous behavioral characterization experiments in the BACHD rat model to keep conditions as much comparable as possible between experiments in the same facility. All experimental animals were maintained in a room with constant temperature (22 ± 1°C) and humidity (55 ± 10%) with a regular 12 h light/dark cycle (lights on/off at 6:00 A.M. /6:00 P.M.).

All experimental animals in both facilities were provided food and water *ad libitum*, and behavioural tests were conducted during the active (dark) phase of the cycle.

The BACHD rat colony in Nijmegen, including the animals used for this study, was the F1 generation of animals ordered from the original breeder that were bred in-house with regular SD wild-type females, also specifically ordered from the regular supplier. Since the same procedure of generating animals for sectional and longitudinal studies has been used in Tuebingen and the two colonies have not been bred on different sites for a long period of time, the chance of genetic drift or other breeding/colony-effects is relatively low. Notably, other studies confirmed that the timeline of onset of deficits, such as the impaired Rotarod performance, is comparable between different labs applying different housing conditions [10–12].

### Behavioral procedures

#### Experiment 1: Social interaction test

**Apparatus and procedures.** A total of 48 male rats (24 WT and 24 BACHD) was used for the study. All animals were longitudinally tested during the dark phase at 2, 4 and 8 months to monitor the progression of deficits and underwent the same schedule. The protocol of the social interaction test (adapted from [21]) is based on social interest and interaction with a familiar animal. Table 1 provides a survey of the timeline of experiments, performed as follows: on day 1, the body weight was measured and a first 20 minute habituation session in the test-arena was given to each animal individually. On day 2 cagemates were habituated (i.e. under social conditions) for a second time to the test arena for another 20 minute session. Any initial novelty-induced behavior declines after repeated exposure to the test environment [22,23] therefore the effect of repeated testing was considered to be minimized by these 2 days of habituation that were repeated for each test at each age-point. On day 3 animals were given 1 day-rest and on day 4 cagemates were separated and individually housed for 24 h. During this period of time cagemates were unable to see each other, but could smell and hear all the animals present in the room. Thus, only real active social interaction was prevented.

**Table 1:** Timeline of experiment 1.

Age	Days						
	1		2	3	4	5	
**2 Months**	**Body Weight**	**Single Habituation**	**Habituation in Pairs**	**Rest day**	**Individual Housing**	**Social Interaction Test**	
**4 Months**	**Body Weight**	**Single Habituation**	**Habituation in Pairs**	**Rest day**	**Individual Housing**	**Social Interaction Test**	
**8 Months**	**Body Weight**	**Single Habituation**	**Habituation in Pairs**	**Rest day**	**Individual Housing**	**Social Interaction Test**	**Animals sacrificed**

The day of rest was given for practical reasons since the testing required the full day without any chance of separating the animals in between. On day 5 cagemates were brought together and tested for 20 min in the social interaction test.

**Fig 1:**
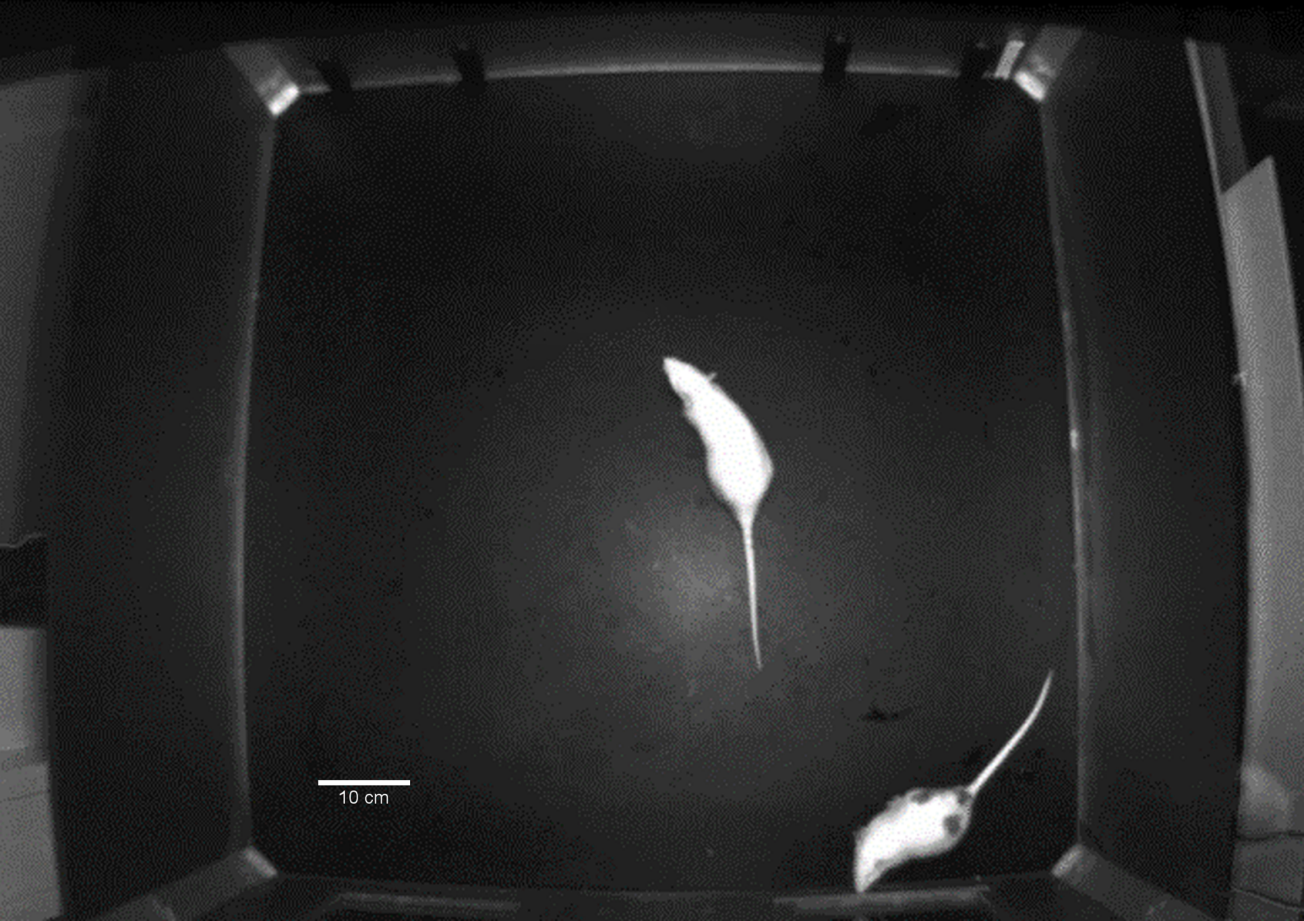
PhenoTyper® 9000 (PT9000) cage setup for testing. A photo displaying two rats during the social interaction test. Cagemates were brought together after being individually housed for 24h. Animals were marked red or black using a permanent marker in order to distinguish each rat of the couple. In contrast to black marking, the red marking was not visible because of the infrared lighting conditions, and it was used to prevent that the marking could become a confounding factor.

The arena used in this experiment consisted of an enlarged, customized version of the PhenoTyper® (PhenoTyper 9000, Noldus Information Technology, Wageningen, The Netherlands). The animals were tested in these large chambers since it is argued that the expression of social behavior requires space [21,24]. The arenas themselves consisted of a black floor plate (floor dimensions: 90 × 90 cm) and transparent Perspex walls (height: 100 cm) (Fig 1). The large PhenoTypers (PT-900) were equipped with a camera and infrared lighting. PhenoTypers were wiped clean with 70% ethanol between test subjects.

**Variables measured.** Video recordings were used to score behavior from captured video files using ½ playback speed of the video by using Observer XT 12.5 (Noldus Information Technology, Wageningen, The Netherlands). The time spent in social interactions (see Table 2 for the used ethogram, adapted from [21]) was manually scored by one observer blind to the test subjects’ genotype. Since the behavioral elements were scored from the view of one of the animals (focal animal) of a pair, behavioral elements from the category ‘social’ are scored either as receiver or as actor [21] and data are thus representative of N = 12 per genotype.

**Table 2:** Ethogram. Definitions of all scored behaviors in the social interaction test.

Behavioral category	Behavioral element	Description
*Social contact*	Nape attacking	The focal animal attacks the neck area of another with its front part of the body
	Pinning	An animal turns on its back and the focal animal pins the other animal to the ground with its forepaws or its whole body
	Social nose contact	The focal animal establishes contact or near-contact with its nose to the another animals’ body part
	Allogrooming	The focal grooming animal has one or both front paws on top of the other and pulls/licks at its fur
*Social interest*	Approaching	The focal animal gets in proximity of another animal, by a directed movement whereby the space between the 2 animals readily decreases
	Following	The focal animal moves/follow the other to maintain a close distance while the other animal is moving around/away
*Social avoidance*	Moving away	The focal animal moves away from another animal after being in close proximity
*Non-social*	Solitary	The animal performs individual actions such as self-grooming, rearing, exploration (at least 1 body-length away from the other animal)

#### Experiment 2: Three chamber social test

**Apparatus and procedure.** A three chamber social test was applied in a cohort of 7 month old rats (17 WT and 15 BACHD) to assess sociability and social novelty using procedures adapted from previously published protocols [25,26]. Behavior was assessed in a black Plexiglas box (40 x 120 x 45 cm) divided in three sub-chambers (or arenas) consisting of a central arena (named neutral arena) and two lateral ones, interconnected by two transparent walls. While the central arena was empty for the whole length of the test, each lateral arena contained a wire mesh box that was either empty or hosted a stranger rat, depending on the testing phase. These stimulus-animals were naïve age- and weight-matched wild-type male rats from the same strain. Restraining the stranger rats in these boxes has the advantage of limiting the mobility of the stranger rat while still allowing visual, olfactory and tactile interaction between the experimental rat and the strangers. By limiting the mobility of the stranger rats, one can examine the social interest of a test animal reducing the influence of the social interaction levels of the strangers in the test performance. In this way, a test animal can choose whether to explore a stranger and whether to explore the familiar or the novel animal that instead serve as social stimuli. Moreover, placing stranger animals in wire mesh boxes prevents the development of possible aggressive responses that may develop between test and stranger animals.

Testing was performed in the dark phase. Animals were allowed to habituate to the testing room for an hour before starting the test that consisted of three consecutive sessions: habituation (5 min), social interaction (10 min, 1 stranger rat present) and social novelty (10 min, 2 stranger rats present). Animals were given a 7 min intertrial interval between test sessions. During the first session (habituation), an experimental rat was placed in the central arena and was allowed to explore the box while each lateral arena contained an empty wire mesh box. In the second session (social interaction), a stranger conspecific rat was placed in the cage of one of the two lateral arenas while the test rat was re-introduced in the neutral arena and let explore the whole box. In this phase the preference of the test rat for exploring the stranger (social stimulus) or an empty box (non-social stimulus) on the opposite side is measured. In the third session (social novelty), a second stranger rat was placed in the cage of the opposite lateral arena. In this phase, the experimental rat can choose whether to explore the rat that was already present in the box in the social interaction phase (familiar rat) or the newly introduced one (novel rat). Social and novelty arenas and strangers were randomized between left and right sides in the box to avoid that a side preference in the box may influence the time spent exploring social/familiar or novelty arenas and relative strangers. The strangers were additionally randomized between WT and BACHD testing animals and between test phases. To prevent that removing animals from a cage could affect the performance of the remaining animals in the same cage, testing of cage mates on the same day was avoided. The stranger animals were not habituated to the wire mesh cages before the test. However, since the order of testing was randomized, as mentioned above, any effect of confinement of the strangers in the boxes is expected to be equally distributed over the groups/genotypes, preventing a strong effect on any genotype-results. The chamber and the wire mesh cages were wiped clean with 70% ethanol between test subjects.

**Variables measured.** The time spent in each arena and the time spent exploring the wire mesh boxes in each phase were manually scored with The Observer XT 12.5 (Noldus Information Technology, Wageningen, The Netherlands). Wire mesh box exploration was defined as sniffing the box (apparent snout contact), climbing the box, resting on the box with the front limbs, sitting and moving on the box. A rat was considered accessing a chamber when entering inside with half of the body length.

#### Reliability analysis

Since behavioral variables were scored manually in both social tests, a second observer re-scored part of the social interaction test and the three chamber social test videos in order to assess the reliability of our results. The second scoring was performed using ½ playback speed of the video with Observer XT 12.5 on six videos (three for each genotype) for each test, randomly chosen. By using a built-in reliability analysis feature in The Observer XT, the percentages of agreement between the two observers were calculated and the resulting statistics is presented in the Supporting Information (S1 and S2 Tables).

#### Tissue collection

After the last social interaction test 8 months-old animals were euthanized by intraperitoneal injection of 90 mg/kg pentobarbital. Immediately after death, the animals were decapitated and their brains removed, isolated and frozen in aluminium foil on dry ice and stored at −80°C. In a cryostat (−12°C), the brains were prepared in 300 μm-thick coronal slices in order to obtain punches from dorsal and ventral parts of the striatum (Bregma +3.72 and +3.30 mm). The brain areas were bilaterally punched out with a Miltex 1.0 mm biopsy puncher (Miltex Inc., York, PA, USA), collected in sterile vials, immediately placed on dry ice and stored at −80°C.

#### RNA extraction

Total RNA was isolated by single step guanidinium isothiocyanate/phenol extraction using PureZol RNA isolation reagent (Bio-Rad, Hercules, USA), according to manufacturer’s instructions. RNA concentrations were measured and RNA purity checked (A260/280 ratio between 1.8 and 2.0) with a NanoDrop 1000 spectrophotometer (Thermo Scientific, Waltham, USA). Subsequently, an aliquot of each sample was treated with DNAse to avoid DNA contamination to perform gene expression analyses as previously reported [22].

#### Real time qRT-PCR

The mRNA levels of total BDNF, D1 and D2 dopamine receptors were analyzed by TaqMan qRT-PCR instrument (CFX384 real-time system, Bio-Rad Laboratories S.r.l) using the iScriptTM one-step RT-PCR kit for probes (Bio-Rad Laboratories S.r.l.). Samples were run in 384-well format in triplicate as multiplexed reactions with a normalizing internal control (β*-actin*). The following primer and probe sequences were used; *Bdnf*-Fw: 5’ AAGTCTGCATTACATTCCTCGA-3’, *Bdnf*-Rev: 5’ GTTTTCTGAAAGAGGGACAGTTTAT -3’, *Bdnf*-Probe: 5’ TGTGGTTTGTTGCCGTTGCCAAG -3’, *D1*-Fw: 5’ GTCTGTCCTTATATCCTTCATCCC -3’, *D1*-Rev: 5’ ATACGTCCTGCTCAACCTTG -3’, *D1*-Probe: 5’ ACAGTTGTCATCCTCGGTGTCCTC -3’, *D2*-Fw: 5’ ACCACTCAAGGGCAACTG -3’, *D2*-Rev: 5’ TGACAGCATCTCCATTTCCAG -3’, *D2*-Probe: 5’ AGCATCCATTCTCCGCCTGTTCA -3’, β*-actin* -Fw: 5’ CACTTTCTACAATGAGCTGCG -3’, β*-actin* -Rev: 5’ CTGGATGGCTACGTACATGG -3’, β*-actin* -Probe: 5’ TCTGGGTCATCTTTTCACGGTTGGC -3’.

Thermal cycling was initiated with a 10-min incubation at 50°C during which the reverse transcription of RNA to cDNA took place (RNA retrotranscription), followed by 5 min at 95°C (TaqMan polymerase activation) and 39 amplification cycles with 10s at 95°C and 30s at 60°C. A comparative cycle threshold (Ct) method was used to calculate the relative target gene expression versus the control group. Specifically, relative target gene expression was calculated according to the 2 ^-ΔCT^ method [23].

### Statistical analyses

Two-way repeated measures ANOVAs were used to analyze all behavioral parameters of the social interaction test. The pairs were the statistical unit, with N = 24 per genotype resulting in 12 pairs of animals per genotype that were analyzed using age as within-subject factor and genotype as between-subject factor. Bonferroni *post-hoc* test was used to follow up any significant effects of genotype found in the two-way ANOVAs. The three chamber social test was analyzed with two-way ANOVA, followed by Tukey or Sidak *post-hoc* test when appropriate. One WT individual was identified as outlier based on the standard deviation of the mean and the residuals of the statistical model and therefore excluded from the statistical analyses. The real time RT-PCR 2 ^-ΔCT^ data have been normalized to the average of the wild-type group and have been analyzed using an unpaired *t* student’s test.

Statistical analyses for the social interaction test and the RT-PCR were conducted using GraphPad Prism v.6.0 (GraphPad Software Inc., San Diego, USA) while analyses of three chamber social test parameters were performed using GraphPad Prism v.7.0. Statistical significance was set at *p*< 0.05 in all tests.

## 4 Results

### Behavioral experiments

#### Experiment 1: Social interaction test

**Fig 2:**
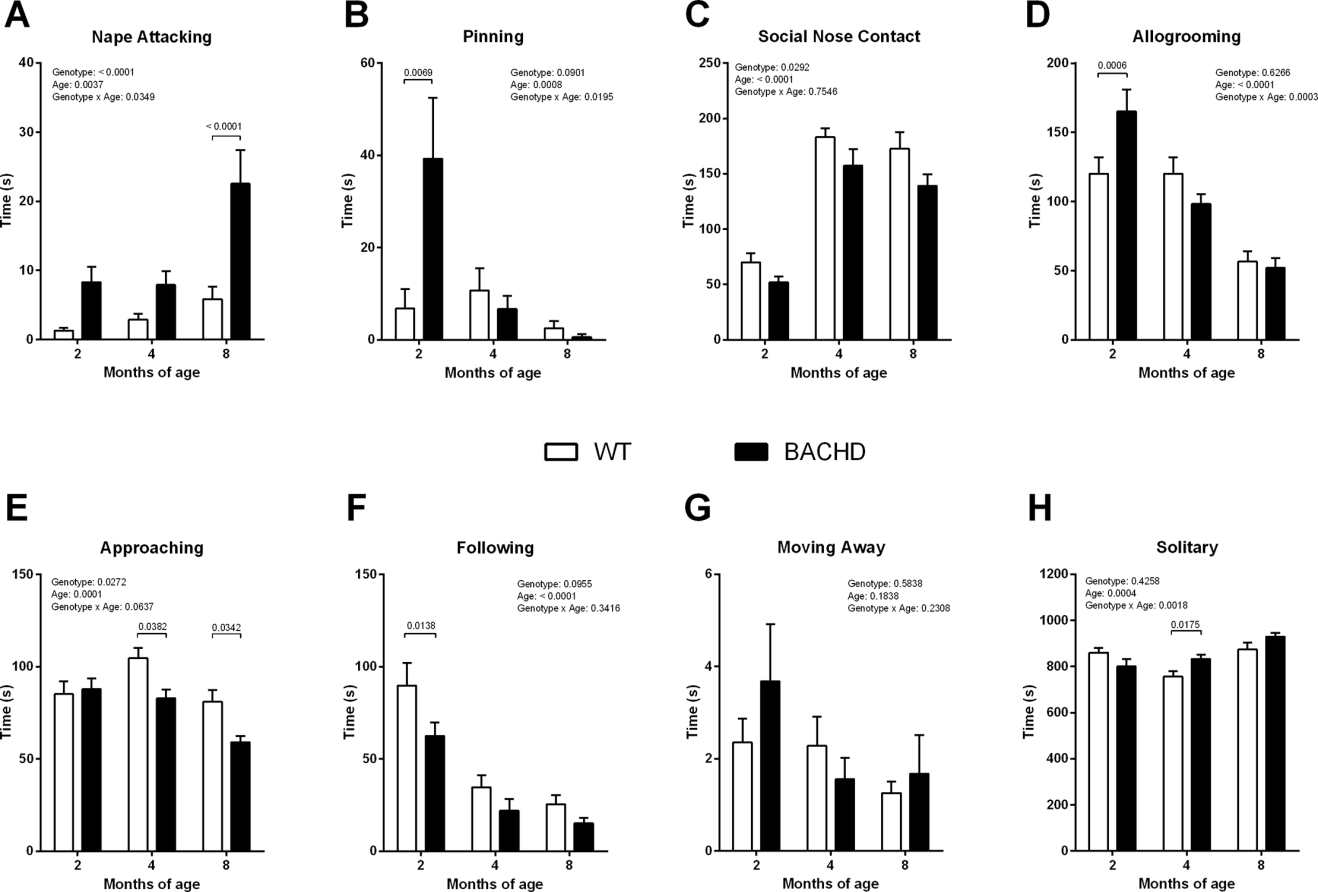
Social interaction test. (A) Nape Attacking. (B) Pinning. (C) Social Nose Contact. (D) Following. (E) Approaching. (F) Allogrooming. (G) Moving Away. (H) Solitary. Data are expressed as means + S.E.M. Two-way ANOVA results are displayed above each graph. Results from *post-hoc* analysis are indicated on the graph in case significant genotype differences were found. N = 12 pairs of WT and 12 pairs of BACHD rats.

Animals of both genotypes showed a significant increase of time spent on ‘‘nape attacking” between 2 and 8 months of age (age effect: F_(2, 22)_ = 7.310, *p* = 0.0037). When compared to their WT littermates, BACHD animals spent an increased time spent on ‘‘nape attacking” showing significant genotype (8 months: *p*<0.0001) and age x genotype (F_(2, 22_) = 3.922, *p* = 0.0349) effects (Fig 2A). Conversely, transgenic animals exhibited a decreased time performing ‘‘pinning” between 2 and 8 months of age, showing a significant genotype (2 months *p*<0.01), age (F_(2, 22)_ = 10.07, *p* = 0.0008) and age x genotype effects (F_(2, 22)_ = 4.733, *p* = 0.0195) (Fig 2B).

‘‘Social nose contact” (sniffing) is the most frequently used parameter to define the interest of an animal in another animal [27]. The duration of ‘‘social nose contact” increased in both WT and BACHD between 2 and 4 months of age while it remained stable between 4 and 8 months of age, showing a significant age (F_(2, 22)_ = 65.78, *p*<0.0001) effect (Fig 2C). At the three different ages BACHD rats spent significantly less time on ‘‘social nose contact” compared to WT rats (genotype effect (F_(1,11)_ = 6.281, *p* = 0.0292).

After an initial increase of “allogrooming” in BACHD rats at 2 months of age (*post-hoc* analyses *p*<0.001) (Fig 2D), with increasing age they exhibited a trend to groom each other less compared to the control rats, showing significant genotype x age effect (F_(2, 22)_ = 11.79, *p* = 0.0003). *Post-hoc* analyses of ‘‘approaching” revealed a significant genotype effect in BACHD rats aged 4 and 8 months (Fig 2E), indicating that transgenic rats showed less “approaching” behavior (*post-hoc* analyses at 4 and 8 months: *p*<0.05). Interestingly, both the “approaching” and “allogrooming” decreased in both BACHD and WT rats between 4 and 8 months of age (significant age effect for approaching (F_(2, 22)_ = 14.02, *p* = 0.0001) and for allogrooming (F_(2, 22)_ = 53.28, *p*<0.0001). Conversely, the “following” significantly decreased in both groups of animals between 2 and 8 months of age (F_(2, 22)_ = 55.02, *p*<0.0001) and *post-hoc* analyses showed significant reduction in the time spent on ‘‘following” of 2 months old BACHD rats compared to WT (2 months: *p*<0.05) (Fig 2F). BACHD rats seemed to spend less time on following the partner compared to the control group. The ‘‘moving away” parameter did not show any significant differences whatsoever (Fig 2G). Concerning non-social behavior, BACHD rats exhibited augmented “solitary” behavior. A significant interaction between age and genotype was found (F_(2, 22)_ = 8.571, *p* = 0.0018) (Fig 2H), although a significant genotype effect was only present at 4 months of age (*post-hoc* analyses *p*<0.05). We further performed a reliability analysis of the present results, and the outcome reported in the Supporting Information (S1 Table).

#### Experiment 2: Three chamber social test

**Fig 3:**
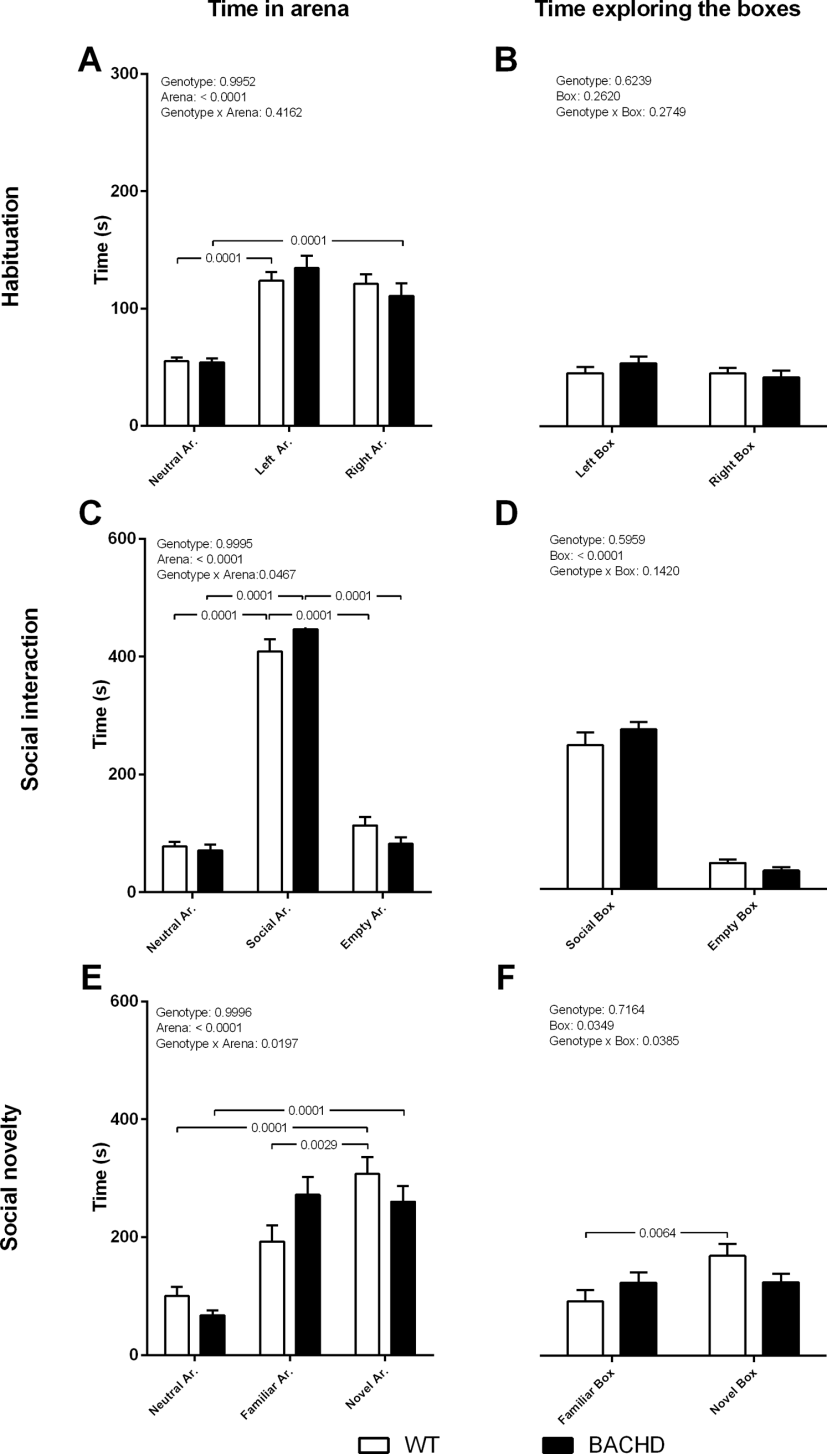
Three chamber social test. The figure shows the time spent in the arenas (A, C, E) and exploring the conspecifics (boxes) (B, D, F) in the three test phases. Data are expressed as means + S.E.M. The p values obtained from the two-way ANOVA analyses, are displayed above each graph. The p values resulting from *post-hoc* analyses are indicated on the graph for significant differences between genotypes as well as for significant differences among arenas and between boxes within genotype. N = 16 WT and 15 BACHD. Abbreviation: Ar. = Arena.

In the habituation phase WT and transgenic animals spent a comparable length of time in the left and right arenas and exploring the empty boxes (Fig 3A and 3B). Within each genotype, no significant preference was detected for left or right arena and the respective empty boxes. Two-way ANOVA showed an arena effect (F_(2,87)_ = 51.92, *p*<0.0001) and no genotype or arena x genotype interaction. Tuckey test revealed only a difference in time spent exploring the neutral arena compared to the lateral compartments (both *p*<0.0001), but no differences between left and right side (*p* = 0.2281).

In the social interaction phase, when a conspecific rat was introduced in one of the boxes, both genotypes showed a comparable preference for the social arena and box with conspecific relative to non-social arena and empty box, respectively (Fig 3C and 3D). Two-way ANOVA in this phase showed an arena effect (F_(2,87)_ = 405.8, *p*<0.0001) and an arena x genotype interaction (F_(2,87)_ = 3.175, *p* = 0.0467), but no genotype differences (F_(2,87)_ = 3.719e^-007^, *p* = 0.9995). *Post-hoc* test indicated a preference for the social arena versus both neutral and empty arenas in both genotypes (all *p*<0.0001). ANOVA analyses showed also a box effect (F_(1,58)_ = 268.6, *p*<0.0001), but no genotype (F_(1,58)_ = 0.2843, *p* = 0.5959) or box x genotype interaction (F_(1,58)_ = 2.216, *p* = 0.1420).

In the social novelty phase, when a novel conspecific was introduced in the previously empty arena, transgenic rats spent a comparable amount of time in the familiar and novel arenas and exploring the boxes with familiar and novel conspecifics, while WT rats stayed longer in the novel than in the familiar arena and showed higher interest in the novel conspecific relative to the familiar one (Fig 3E and 3F). Two-way ANOVA analyses showed an arena effect (F_(2,87)_ = 36.83, *p*<0.0001) and an arena x genotype interaction (F_(2,87)_ = 4.109, *p*<0.05), but no genotype differences (F_(2,87)_ = 2.549e^-007^, *p* = 0.9996). Similarly, ANOVA analyses showed a box effect (F_(1,58)_ = 4.4665, *p* = 0.0349) and a box x genotype interaction (F_(1,58)_ = 4.484, *p =* 0.0385), but no genotype effects (F_(1,58)_ = 0.1332, *p* = 0.7164). *Post-hoc* analyses revealed a significant difference in time spent between novel arena and neutral arena in both genotypes (both *p*<0.0001) and a difference in time spent between novel arena and familiar arena in WT (*p* = 0.0028), but not in transgenic animals (*p* = 0.9390). In line with the arena effects, Sidak test also indicated a significant difference in time spent exploring the novel and familiar conspecific in WT (*p =* 0.0064) and not in transgenic animals (*p* = 0.9995). We further performed a reliability analysis of the present results, and the outcome reported in the Supporting Information (S2 Table).

### Molecular analysis

#### Gene expression analysis of dopamine D1 and D2 receptors in the striatum

**Fig 4:**
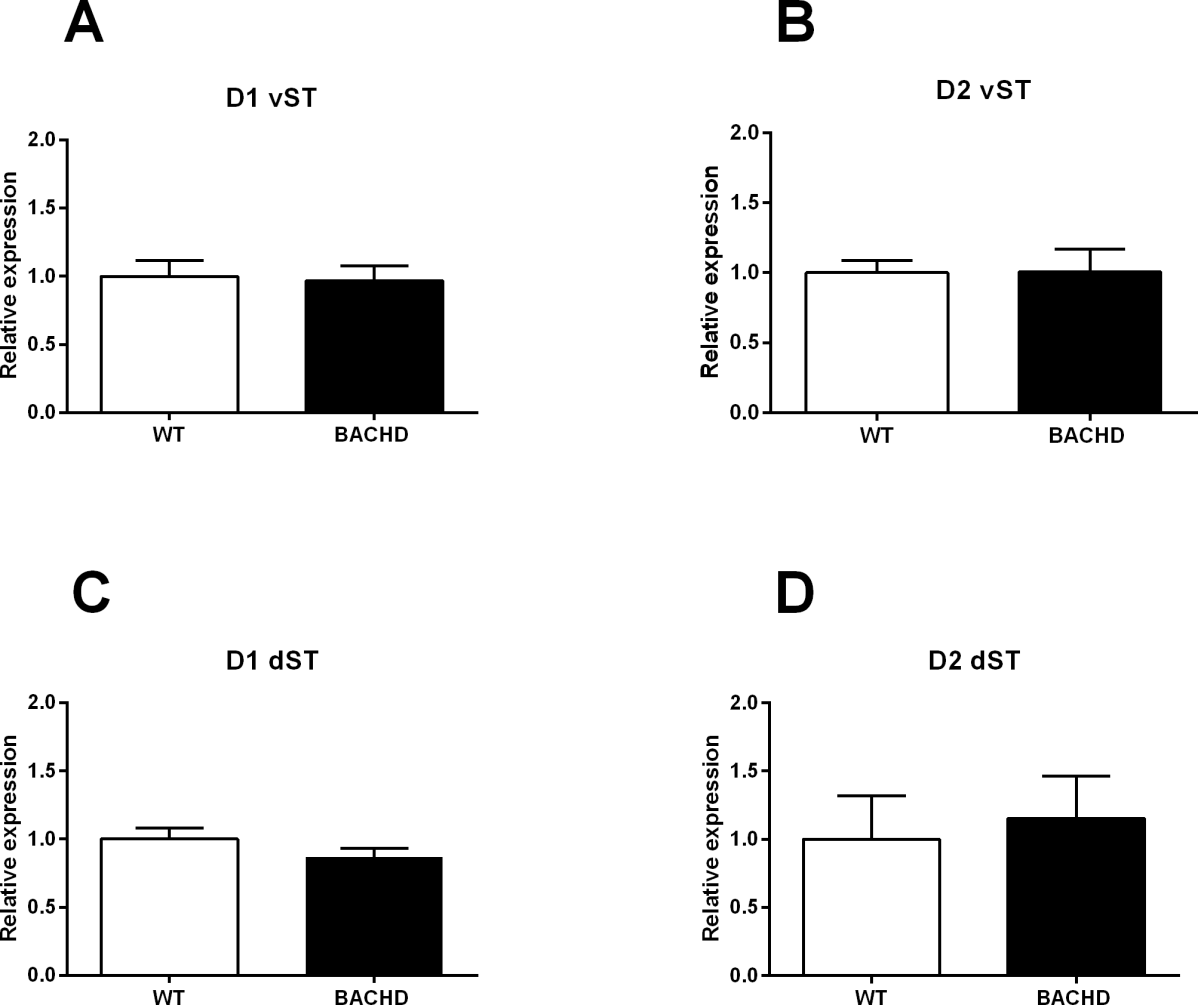
**mRNA levels of D1 (A) and D2 (B) receptors in the ventral striatum and mRNA levels of D1 (C) and D2 (D) receptors in the dorsal striatum of WT and BACHD rats.** Data were normalized to the average of the fold changes in the WT group (set at 1.0) and analyzed using unpaired *t* student’s tests. Abbreviations: vST = ventral striatum; dST = dorsal striatum.

mRNA levels of D1 and D2 dopamine receptors have been measured in the ventral and dorsal striatum of BACHD rats to evaluate their potential involvement in the previously observed behavioral alterations. However, as shown in Fig 4, we did not find any significant difference in the gene expression of both receptors in the mutant animals in comparison with wild-type rats, neither in the ventral (D1: WT 1 ± 0.11, N = 11; BACHD 0.97 ± 0.11, N = 9; t(18) = 0.1947, *p* = 0.8478. D2: WT 1 ± 0.09, N = 11; BACHD 1.01 ± 0.16, N = 9; t(18) = 0.045, *p* = 0.9649) nor in the dorsal striatum (D1: WT 1 ± 0.081, N = 9; BACHD 0.87 ± 0.06, N = 10; t(18) = 1.304, *p* = 0.2096. D2: WT 1 ± 0.1, N = 11; BACHD 1.15 ± 0.09, N = 12; t(21) = 1.175, *p* = 0.2531).

#### Gene expression analysis of BDNF in the striatum

**Fig 5:**
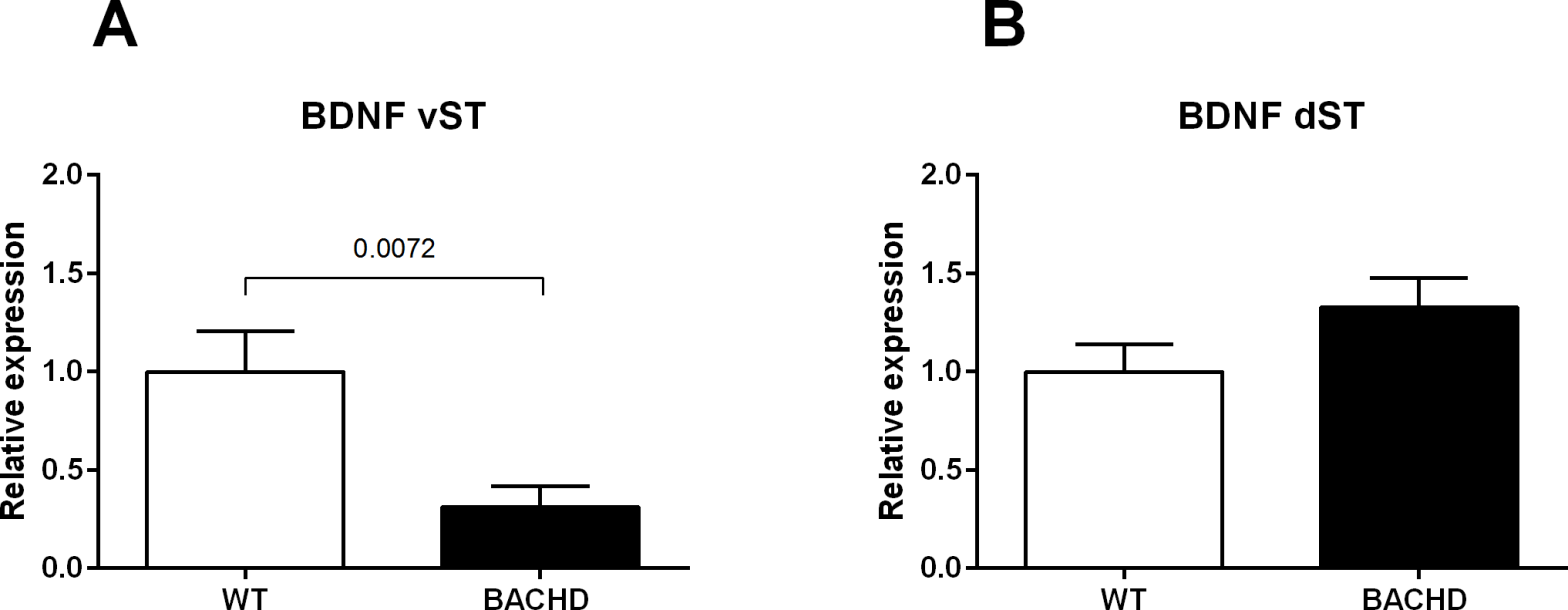
**mRNA levels of total BDNF in the ventral (A) and dorsal (B) striatum of WT and BACHD rats.** Data were normalized to the average of the fold changes in the WT group (set at 1.0). Data were analyzed using unpaired *t* student’s tests. Abbreviations: vST = ventral striatum; dST = dorsal striatum.

Alterations of the neurotrophin Brain-derived neurotrophic factor (BDNF) are thought to be relevant for the neurodegeneration observed in Huntington’s disease [28]. Therefore, we evaluated the mRNA levels of the total form of BDNF in both ventral and dorsal striatum of BACHD rats with respect to wild-type animals. As shown in Fig 5, we observed a significant and marked decrease of the mRNA levels of the total form of BDNF in the ventral striatum of BACHD rats compared to WT littermates (WT 1 ± 0.21, N = 9; BACHD 0.31 ± 0.1050, N = 8; t(15) = 2.846, *p*<0.01). Conversely, the expression of the neurotrophin was not modulated by the genotype in the dorsal striatum (WT 1 ± 0.14, N = 10; BACHD 1.33 ± 0.15 N = 10; t(18) = 1.601, *p* = 0.1269).

## 5 Discussion

The use of multiple social paradigms permitted us to detect potential HD-related deficits in different specific aspects of social behavior. The social interaction test revealed abnormal social play and aggressive behavior in BACHD animals as well as a trend towards a decreased interaction with conspecifics. It is worth mentioning the fact that we decided to use familiar pairs (cagemates) to have a better translational value of the results since HD causes major disruption in family life [29]. The three chamber social test showed mild deficits in social recognition in transgenic animals, providing an estimation of the social interest towards an unknown conspecific as well as of the recognition abilities between familiar and novel conspecifics, with limited physical interaction. Additionally, striatal expression of D1, D2 receptors and BDNF was assessed and related to social contact and social interest in 8 month old animals, reporting a decrease in the expression of BDNF in the ventral striatum and intact dopamine receptor expression.

In rats, one of the most notable social behaviors is play fighting, which involves attack and defense of the nape of the neck, which if contacted, is gently nuzzled with the snout [27]. When initiating social play, one animal directs to the neck region of the partner and this can be accompanied with biting and pulling fur in that region. BACHD rats performed more play-fighting compared to their WT littermates, as showed by the increased nape attacking. 8 month old BACHD rats showed a twofold increase in the time spent on nape attacks, and a decreased duration of pinning compared with control rats. Pinning is a commonly used measure for play, which essentially involves the subject positioned supine with its partner standing on top [27,30].

These rougher and more aggressive play behaviors likely reflect abnormal social play behaviors and might facilitate the development of social and aggressive behaviors [31]. Conversely, “following” significantly decreased in both groups of animals between 2 and 8 months of age, suggesting a reduced inclination to search for an interaction over time. The lack of these social behaviors in BACHD rats may suggest a “social deficit” that could be related to coping styles [32,33], and considered as a form of apathy [17] as showed by the R6/2 mouse model of HD.

Although we cannot directly confirm apathy with the parameters we measured, apathy has been previously reported in the BACHD and in z_Q175 mouse models [34] and is commonly reported in patients with HD [35]. Future studies on other specific aspects of social behavior, e.g., social reward, will be important to define whether or not a social apathy-like phenotype is present in BACHD rats. Alternatively, the deficits in social behaviors described in BACHD rats may represent behavioral changes that depend more specifically on alterations in brain social networks which mechanisms in HD are still mostly unclear.

Interestingly, at 2 months of age the BACHD rats “nape attacking” was significantly increased as well as “pinning”, where we can assume that the rats established the dominance hierarchy, leading to a dominant and a subordinate rat. This disappeared at 4 and 8 months of age, probably because the dominance was already determined.

The lower approaching and following behaviors observed in this study are not likely to be affected by anxiety levels in BACHD rats, as previous studies in this model showed decreased exploratory anxious behavior in the elevated plus maze starting at 4 months of age [10]. If we consider the time spent on nape attacks related to social dominance and dominance to be dependent on anxiety levels [36] with dominant individuals being less anxious than subdominant ones, than the increase in nape attacking in BACHD rats could be facilitated by the lower anxiety levels in these animals. However, one should keep in mind that the decreased anxious-like behavior in BACHD rats was shown only with the elevated plus maze [10]. It is therefore difficult to conclude anything about the anxious phenotype in BACHD rats and to relate it to social behavior parameters.

It is worth noting that these social behavior alterations were not confounded by an overall reduced motor activity, such as levels of general exploration or activity, as BACHD animals reported similar levels of locomotion compared to their wild-type littermates in our previous study [37]. Furthermore, during all other social tests, transgenic animals did not differ from wild-types in the expression of non-social activities.

Besides the changes in social interaction parameters, we also detected a mild deficit in social novelty in transgenic animals at 7 months of age, as suggested by a lack of preference for a novel conspecific relative to a familiar one in the three chamber social test. Cognitive deficits have already been described in BACHD rats for different aspects not related to social behavior [12–14]. The novelty effect in our study is in line with earlier research in HD mouse models showing alterations in social recognition and memory [18,19] as well as with evidence of disrupted social cognition in HD patients [38,39]. The brain changes underlying these symptoms in HD are not well known and it is not clear to which extent deficits in social cognition, mostly described as impaired emotion recognition, may depend on other cognitive and emotional changes and whether they could be the cause of social behavior alterations in HD patients.

While decreased or increased social interaction [15–17] have been described in other animal models of HD using different behavioral paradigms, we did not observe altered sociability levels in BACHD rats exposed to the three chamber social test. In line with these findings, one recent study showed no changes in the preference for an unknown conspecific relative to an unknown object in BACHD rats [40]. Although unchanged levels of social interaction in the three chamber social test are present, they are not necessarily in contrast with parameters measured in the social interaction test within this study. For example, increased approaching in the social interaction test is present along with an increased nape attacking which may relate to a play fighting-like behavior that is prevented in the other test where limited physical contact is allowed.

Gene expression analyses were carried out in 8 month old animals tested for social interaction, highlighting an altered expression of BDNF in ventral striatum and an intact striatal D1 and D2 receptors expression. The striatum is one of the most affected brain areas in HD where alterations in dopaminergic and BDNF brain systems have been well described previously [28,41]. mRNA levels of striatal BDNF and dopaminergic receptors were therefore examined as potential mechanisms at the base of behavioral abnormalities. Furthermore, we linked social behavior to striatal functionality via D1 and D2 dopamine receptors in 8 month old animals which, along with BDNF, have been proposed as possible underlying regulatory factors [42]. The lack of changes in mRNA levels of D1 and D2 dopamine receptors in BACHD rats subjected to the social interaction test would not suggest a direct link between the dopaminergic system and the behavioral changes. Measuring protein levels in future social interaction studies may help to understand better whether the dopaminergic system plays a role in the behavioral changes. BDNF mRNA levels were also quantified. We detected a decreased expression in the ventral striatum of BACHD animals and no changes in the dorsal region. Therefore, it is possible that altered BDNF expression in this region may have contributed to the deficits in sociability in 8 months old BACHD rats. Nonetheless, we are aware that the limit of molecular investigations is the restriction of the analyses to the transcript levels. Striatal BDNF transcripts are very low at basal level and usually BDNF protein is not directly transcribed in the striatum, but it is produced in the cerebral cortex and anterogradely transported to the striatum [43]. Therefore, we cannot draw any conclusion on the mechanisms underlying the BDNF decrease and its link to social deficits in BACHD rats and it will be assessed in future experiments. Alternative mechanisms that could have contributed to the social deficits and are worthwhile to assess in future studies are the oxytocinergic and vasopressinergic systems, which are interesting in the context of their involvement in social recognition and social behavior [42].

The strength of our study is that we performed two different behavior experiments to investigate different aspects of BACHD rats’ social behavior: the social interaction test was performed longitudinally to make the monitoring of behavior across different ages possible; the three chamber social test provided an estimation of the social interest towards an unknown conspecific as well as of the recognition abilities between familiar and novel conspecifics. Additionally, two different observer coded the behaviors in both social tests, strengthening the reliability of our results. Accordingly, the correspondence is in line with inter-observer agreement, i.e., generally acceptable to a level of 70–85% [44]. On the other hand, we are aware that animals of both experiments had different housing conditions and applying the same housing conditions in the two experiments would have improved the study design since they can affect animals’ behavior. However, this was not the main goal of this chosen setup, and we do not consider our results incompatible, since we should bear in mind that: 1) this study assesses different aspects of social behavior in the same animal model and does not present a direct comparison of the same parameters obtained with two different tests; 2) we do not make any statistical correlation of the changes in parameters obtained in one test with those shown in the other test; 3) we do not think that the results shown in the social interaction test are in contrast to those in the three chamber social test. We do not know to what extent the housing conditions may have affected the results in each experiment of our study because these tests were performed for the first time in the BACHD rat model. Performing both experiments with each of the housing settings would be certainly informative about that although this was not the aim of the present study. In conclusion, this study characterizes social behavior in the BACHD rat model. We report changes in different parameters of social interaction and recognition and potential molecular correlates by using paradigms that are well established in rodents [31,45] and that can be used to study disturbances in this spectrum [45]. Being easily measurable, such parameters provide the basis for further social behavior studies and may represent valid readouts for treatment studies.

## Supporting information

S1 Table Percentage of scoring agreement–Social Interaction Test. (DOCX)

S2 Table Percentage of scoring agreement–Three Chamber Social Test. (DOCX)
