# Supplementary material for: BACHD rats expressing full-length mutant huntingtin exhibit differences in social behavior compared to wild-type littermates
Source: PLoS One. 2018 Feb 7;13(2):e0192289. doi: 10.1371/journal.pone.0192289 (PMC5802907; doi:10.1371/journal.pone.0192289)
Supplement: S1 Table — (DOCX) [file pone.0192289.s001.docx]

**S1 Table. Percentage of scoring agreement – Social Interaction Test.**

| **Genotype** | **Percentage of agreement ± SEM** |
| --- | --- |
| WT | 77.396 ± 1.902 |
| BACHD | 82.188 ± 1.042 |

Data are expressed as means ± S.E.M
