# Supplementary material for: BACHD rats expressing full-length mutant huntingtin exhibit differences in social behavior compared to wild-type littermates
Source: PLoS One. 2018 Feb 7;13(2):e0192289. doi: 10.1371/journal.pone.0192289 (PMC5802907; doi:10.1371/journal.pone.0192289)
Supplement: S2 Table — (DOCX) [file pone.0192289.s002.docx]

**S2 Table. Percentage of scoring agreement – Three Chamber Social Test.**

| Genotype | Test Phase | Percentage of agreement ± SEM |
| --- | --- | --- |
| WT | Habituation | 89.620 ± 0.828 |
|  | Social Interaction | 90.800 ± 3.933 |
|  | Social Novelty | 89.234 ± 1.096 |
| BACHD | Habituation | 95.041 ± 0.463 |
|  | Social Interaction | 91.283 ± 3.072 |
|  | Social Novelty | 92.189 ± 1.722 |

Data are expressed as means ± S.E.M
